# Case of Paralysis of the Kidneys

**Published:** 1822-02

**Authors:** John Tweeddale

**Affiliations:** Surgeon.


### Art. IV.—*Case of Paralysis of the Kidneys.*

By John Tweeddale, Surgeon.

MRS. ELIZABETH FULLER, aged fifty-two years, a corpulent woman, had enjoyed a good state of health until the last two months, when she complained of great difficulty of breathing, after taking exercise, accompanied with slight cough. She had borne a child at an early age, and the catamenia had ceased when she was about forty-six years old. In the afternoon of Friday, the 3d of August, while at dinner, she was seized, suddenly, with severe pain in the forehead, accompanied with faintness and difficult respiration. The natural colour of her countenance, and other parts of her body, changed to that of a leaden hue, and her lips became livid. The skin felt cold and moist; the pulse slow and oppressed; the tongue clean; the eyes were suffused with blood; and she felt much inclined to coma. In this state she continued until the next day, when I first saw her. A blister had been applied to the sternum, and she had taken an aperient mixture, by the advice of her medical attendant; but without benefit, or procuring any evacuation from the bowels. It was reported to me, that no urine had been passed since the preceding day at noon; yet no pain was excited, neither did she make any complaint of a sense of fullness, in either the regions of the bladder or of the kidneys, when pressure was made on them with the hand, although cautiously increased to a degree as to feel, distinctly, the pulsation of the abdominal aorta. I remarked that the patient breathed easily, and she seemed to suffer from no pain. The countenance was decidedly swollen; the colour of the body and lips remain as before; the skin was cold, and constantly moist from increased perspiration. The blister has risen, and proper dressing was ordered for it. Warm fomentations were desired to be applied to the extremities from time to time, and a little warm wine and water to be given. She took calomel gr. iv. et pil. aloes c. gr. viii., with an aperient mixture, consisting of the sulphate of magnesia and infusion of senna.

At 6 o'clock P. M. she had two black-coloured stools, but without voiding a single drop of urine. Occasional vomiting of a dark-coloured fluid, without smell, supervened. She feels no inclination for food, but drinks plentifully.

℞. Sp. ætheris nitrosi, f.ʒvj.

Sp. lavend. c. f.ʒiv.

Syr. communis, ʒvij.

Aq. menth. sativ. f.℥viij.

F. M. cujus capiat cochlearia duo majora secundis horis.

*August 5th.*—Has passed a quiet night, with sleep, and has had two more of the same coloured stools, without, however, passing a drop of urine. Still she makes no complaint of pain or fullness in the region of the bladder.—Cont. omnia, et rept. pil. ex calomel et aloe c. una cum mistura aperiente.

*6th.*—Has passed no urine. She complains of a sense of oppression across the chest. No alvine evacuation. The skin feels colder, and very moist; pulse weak and feeble. Continues sensible. Voice very indistinct; the countenance and neck appear much swollen; the colour of the body and lips as before; breathes easily. In this state she began gradually to sink, until about twelve o'clock, when she expired.

Neither the attendants nor myself could perceive any urinous smell; but they stated that there was a disagreeable earthy odour about her. The body, after death, returned to its natural colour; and, on examining it, the bladder was found perfectly empty, yet quite healthy. The kidneys appeared somewhat smaller than natural in size. The cortical portion was of a looser texture, and of a much paler colour, than it has generally. All the other abdominal viscera were healthy. There was a slight effusion of serum in the cavity of the chest, to the amount of between two and three ounces; but neither the lungs nor the heart exhibited the least symptom of disease.

*Upper St. Martin's-lane; December* 1821.

